# Between Patient Care and Personal Safety During Missile Attacks: A Real-Time Participatory Action Research Study of Moral Injury among Healthcare Workers in Long-Term Care Settings

**DOI:** 10.1192/j.eurpsy.2026.10976

**Published:** 2026-07-31

**Authors:** S. Toker, A. Shirazki, I. Shugaev, Y. Tamarkin

**Affiliations:** 1 Organizational Behaviour, Tel Aviv University, Tel Aviv; 2 management, Fliman Geriatric Hospital, Haifa, Israel

## Abstract

**Introduction:**

Healthcare workers in northern Israel faced impossible choices during missile attacks: abandon vulnerable patients or risk their lives. Moral injury refers to psychological consequences following violations of deeply held moral beliefs (Litz et al. Clin Psychol Rev 2009; 29 695-706). No research has examined civilian healthcare moral injury during warfare.

**Objectives:**

**Primary:** Characterize war-related moral injury prevalence, hypothesizing exposure to potentially morally injurious events (PMIEs) associates with moral injury symptoms . **Secondary:** Establish pathways from PMIEs to depression, burnout, performance impairment, and turnover intentions, hypothesizing moral injury mediates these relationships. **Tertiary:** Identify protective factors (resilience, organizational support) that moderate PMIE-to-moral injury and moral injury-to-outcomes pathways.

**Methods:**

This mixed-methods study employed participatory action research at a 370-bed geriatric rehabilitation hospital in northern Israel during the Israel-Hamas conflict (August-November 2024).

**Phase 1: Qualitative Investigation** captured real-time experiences through semi-structured interviews with 12 participants (3 males, 9 females): physicians (n=2), nurses (n=5), and healthcare managers (n=5).

**Phase 2: Quantitative Assessment** involved hospital-wide survey administration three weeks post-ceasefire. Of 370 employees, 198 completed surveys (53% response rate). Sample comprised 100 females (50%), 79 nurses (40%), 24 physicians (12%).

**Results:**

Qualitative analysis revealed Ministry of Health evacuation protocols created potentially morally injurious events for healthcare workers.

**Universal PMIE Exposure:** All 12 participants experienced situations meeting PMIE criteria during missile attacks

**Infrastructure as Moral Hazard:** Physical limitations of protected spaces created systematic moral conflicts

**Role-Specific Impacts:** Direct patient care providers showed highest frequency of moral distress themes

**Social Dimension:** Patient and family awareness/criticism amplified moral injury potential

**Time Pressure Factor:** 60-second evacuation window eliminated possibility for ethical deliberation

**Relationship Context:** Long-term care setting intensified sense of betrayal and abandonmen Complementary quantitative analysis revealed significant associations between moral injury incidence and burnout and depression symptoms, as well as impaired work performance and leave intentions. Resilience anf colleague support had protective effects, whereas managerial support did not.

**Conclusions:**

These findings highlight the urgent need for healthcare systems to redesign emergency protocols and physical infrastructure to prevent morally injurious conflicts while strengthening organizational support mechanisms to protect healthcare worker wellbeing during crisis situations.

**Disclosure of Interest:**

None Declared

